# Fluorodeoxyglucose-positron emission tomography/computed tomography imaging features of colloid adenocarcinoma of the lung: a case report

**DOI:** 10.1186/s13256-017-1380-6

**Published:** 2017-07-27

**Authors:** ZhenGuang Wang, MingMing Yu, YueHua Chen, Yan Kong

**Affiliations:** 1 PET/CT Center, The Affiliated Hospital of Qingdao University, No. 59, Haier Rd, Qingdao, 225001 China; 2 Intense care unit, The Affiliated Hospital of Qingdao University, Qingdao, China

**Keywords:** Adenocarcinoma, Mucinous, Lung neoplasms, Positron emission tomography, Computed tomography

## Abstract

**Background:**

Colloid adenocarcinoma of the lung is a rare subtype of variants of invasive adenocarcinomas. We report the appearance of this unusual entity on ^18^F-fluorodeoxyglucose positron emission tomography/computed tomography.

**Case presentation:**

A 60-year-old man of Chinese Han nationality coughed with a little white sputum for 1 month. Chest computed tomography showed multiple bilateral subpleural nodules and plaques accompanied by air bronchograms, which were most concentrated in the lower lobe of his right lung. Positron emission tomography indicated increased radioactivity uptake with a maximum standardized uptake value of 3.5. Positron emission tomography/computed tomography showed a soft tissue density lesion in his left adrenal gland with a maximum standardized uptake value of 4.1. The positron emission tomography/computed tomography appearance suggested a primary colloid adenocarcinoma in the lower lobe of his right lung accompanied by intrapulmonary and left adrenal gland metastases. The diagnostic rate of colloid adenocarcinoma can be increased by combining the anatomic and metabolic information of lesions.

**Conclusions:**

The advantage of positron emission tomography/computed tomography in the diagnosis of colloid adenocarcinoma, as with other cancers, is the ability to locate extrapulmonary disease, facilitating clinical staging.

## Background

Colloid adenocarcinoma, a subtype of variants of invasive adenocarcinomas, is characterized histologically by abundant mucus in the tumor [1]. Colloid adenocarcinoma accounts for 0.24% of all lung cancers [2] and has a 5-year survival rate of 51% [3]. Our understanding of colloid adenocarcinoma is insufficient owing to its low incidence in clinical practice. A deeper understanding of the imaging manifestations of colloid adenocarcinoma would be of great significance for the diagnosis and treatment of this disease.

**Fig. 1:**
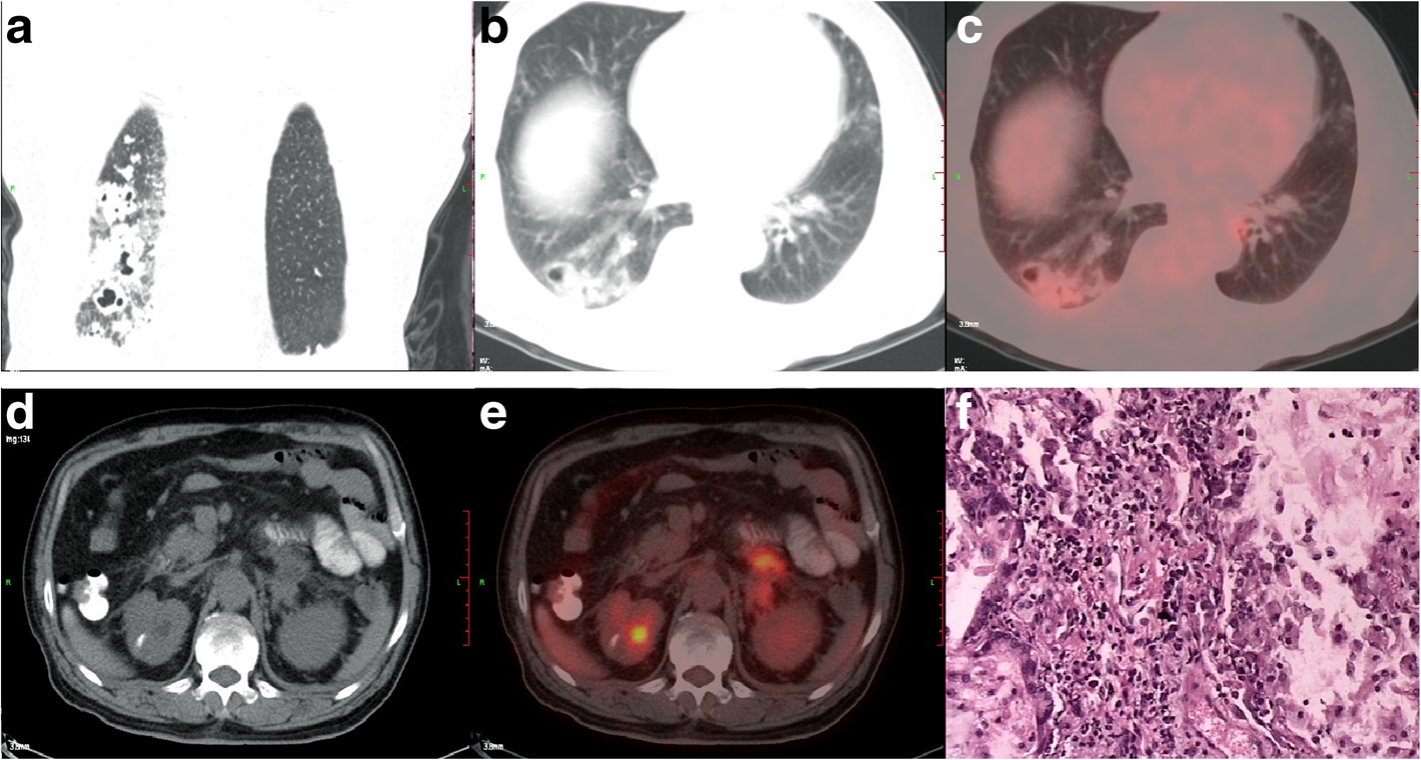
A 60-year-old man with colloid adenocarcinoma. Coronal (**a**) and axial (**b**) chest computed tomography images showed irregular subpleural nodules and plaques accompanied by air bronchograms that involved the lower lobe of the right lung. Positron emission tomography/computed tomography showed there was slightly increased radioactivity uptake, with maximum standardized uptake value of 3.5 (**c**). A soft tissue density lesion was found in the left adrenal gland (**d**) with increased radioactivity uptake and maximum standardized uptake value of 4.1 (**e**). On histological examination, the tumor was composed predominantly of pools of mucin; hematoxylin and eosin stain, ×400 (**f**)

## Case presentation

A 60-year-old man of Chinese Han nationality coughed with a little white sputum for 1 month. A physical examination showed coarse breath sounds in bilateral lungs with moist rales in the lower lobe of his right lung. The tumor marker cancer antigen 125 (CA-125) was 45.84 U/ml (0 to 35.0) and cytokerantin-19-fragment (CYFRA21-1) was 17.79 ng/ml (0 to 3.3). A chest computed tomography (CT) scan showed multiple plaques and nodules with air bronchograms in bilateral lungs; infectious lesions were suspected.

He fasted for at least 6 hours before an intravenous injection of ^18^F-fluorodeoxyglucose (^18^F-FDG) at a dose of 0.2 mCi/kg. After resting for 60 minutes, he was given 300 to 500 mL of pure drinking water followed by bladder emptying before a positron emission tomography (PET)/CT examination (Discovery® VCT; GE Healthcare, Milwaukee WI, USA). Before PET scanning, full body CT scanning from his skull base to his mid-femurs was acquired for attenuation correction and anatomic localization with the following scanning parameters: voltage, 120 kV; current, 130 mA; slice thickness, 3.75 mm; and interlayer spacing, 3.27 mm. The PET scanning conditions were as follows: during quiet breathing, the scan time for each bed position was 3 minutes. For the diagnostic chest CT scan, he was trained in breath holding before the examination, and the scan was conducted from his thoracic inlet to the level of his adrenal glands with inspiratory breath hold [4].

Irregular subpleural nodules and plaques accompanied by air bronchograms involved the lower lobe of his right lung (Fig. 1a, b). There was slightly increased radioactivity uptake, with maximum standardized uptake value (SUV_max_) of 3.5 (Fig. 1c). Multiple irregular nodules accompanied by air bronchograms involved the subpleural regions of bilateral lungs with slightly increased radioactivity uptake and SUV_max_ was 1.7. A soft tissue density lesion was found in the left adrenal gland with increased radioactivity uptake and SUV_max_ was 4.1 (Fig. 1﻿d, e).

He underwent transthoracic needle biopsy of the lesion of the lower lobe of his right lung. On histological examination, the lesion was composed predominantly of pools of mucin and we suspected colloid adenocarcinoma (Fig. 1f). CDX2, MUC2, CK7, and TTF-1 were positive in this case.

## Discussion

Colloid adenocarcinoma originates from columnar epithelial cells or goblet cells, and can secrete a large amount of mucus, resulting in increased mucus in the alveoli [5]. In 2004, Rossi *et al*. summarized the imaging manifestations of 18 cases of colloid adenocarcinoma, and concluded that the most common imaging manifestation was ill-defined solid peripheral nodules (eight cases) [2]. Other manifestations included consolidation accompanied by air bronchograms and ground-glass opacities (six cases and four cases, respectively) [2]. A study conducted by Sawada *et al*. also showed solid or partially solid nodules with air bronchograms [6]. The chest CT in this case showed multiple bilateral subpleural nodules and plaques accompanied by air bronchograms, most concentrated in the lower lobe of our patient’s right lung. The potential pathological basis is that the tumor cells grew along the alveolar and bronchial walls and a one-way valve effect caused the alveoli to distend with air, resulting in air bronchograms [7].

The PET in this case showed that the radioactivity uptake of the lesions in our patient’s bilateral lungs and his left adrenal gland was slightly increased with SUV_max_ of 3.5 and 4.1, respectively. We suspected colloid adenocarcinoma in the lower lobe of his right lung with intrapulmonary and left adrenal gland metastases. The radioactivity uptake of colloid adenocarcinoma was relatively lower. This may be due in part to: (1) large amounts of mucus in the tumor; (2) and the metabolism of slow-growing tumors is generally low [8].

Colloid adenocarcinoma has a higher frequency of metastasis than other types of lung cancer, including intrapulmonary metastasis, hematogenous metastasis, and lymphatic metastasis [9]. Studies have shown that colloid adenocarcinoma tends to spread along the airway mucosa and results in intrapulmonary metastases [10]. A follow-up study conducted by Oka *et al*. for 13 cases of colloid adenocarcinoma confirmed after surgery or biopsy indicated that primary tumors with diameters ≤3 cm had a better prognosis, while larger tumors tended to recur and develop intrapulmonary metastases [11]. This case indicated that the colloid adenocarcinoma from the lower lobe of our patient’s right lung metastasized to bilateral lungs and his left adrenal gland. PET/CT combined with the diagnostic chest CT were valuable for diagnosis and staging of colloid adenocarcinoma although colloid adenocarcinoma had a relatively lower FDG uptake.

## Conclusions

Proper staging is of significant value for the evaluation of patient prognosis as well as the selection of treatment protocols. PET/CT combined with a diagnostic chest CT were valuable for diagnosis and staging of colloid adenocarcinoma although the colloid adenocarcinoma had a relative lower FDG uptake.

## Abbreviations

^18^F-FDG: ^18^F-fluorodeoxyglucose
CT: Computed tomography
PET: Positron emission tomography
SUV_max_: Maximum standardized uptake value.
